# Identifying Drug-Resistant Tuberculosis in Chest Radiographs: Evaluation of CNN Architectures and Training Strategies

**DOI:** 10.1109/EMBC46164.2021.9630189

**Published:** 2021-11

**Authors:** Manohar Karki, Karthik Kantipudi, Hang Yu, Feng Yang, Yasmin M. Kassim, Ziv Yaniv, Stefan Jaeger

**Affiliations:** 1 Lister Hill National Center for Biomedical Communications, U.S. National Library of Medicine, Bethesda, MD 20894 USA; 2 Office of Cyber Infrastructure and Computational Biology, National Institute of Allergy and Infectious Diseases, Bethesda, MD 20894 USA

## Abstract

Tuberculosis (TB) is a serious infectious disease that mainly affects the lungs. Drug resistance to the disease makes it more challenging to control. Early diagnosis of drug resistance can help with decision making resulting in appropriate and successful treatment. Chest X-rays (CXRs) have been pivotal to identifying tuberculosis and are widely available. In this work, we utilize CXRs to distinguish between drug-resistant and drug-sensitive tuberculosis. We incorporate Convolutional Neural Network (CNN) based models to discriminate the two types of TB, and employ standard and deep learning based data augmentation methods to improve the classification. Using labeled data from NIAID TB Portals and additional non-labeled sources, we were able to achieve an Area Under the ROC Curve (AUC) of up to 85% using a pretrained InceptionV3 network.

## I. INTRODUCTION

Tuberculosis (TB) is a global disease caused by the bacterium *Mycobacterium tuberculosis*, which is spread through the air. According to the World Health Organization, in 2019 an estimated 10 million people were infected with TB and about 1.4 million died from the disease [1]. Efforts to control TB have been hindered by the rise of drug-resistant strains, where in 2019 about half a million people developed rifampicin-resistant TB out of which 78% were multidrug-resistant [1]. Early detection of drug resistance enables more specific drug treatment, reduces the period of infectiousness and disease spread in addition to improving outcomes [2].

Current diagnostic methods for identifying drug-resistant TB (DR-TB) infections include conventional culture growth over several weeks and rapid molecular testing [3]. These procedures are not feasible globally, especially for countries unable to scale up their testing capacities. An automated computational approach that utilizes widely available technology is thus desirable. Chest X-rays (CXRs) are extensively used in detection of tuberculosis, and thus offer a potentially natural avenue for discriminating between DR-TB and drug-sensitive TB (DS-TB).

In this work, we evaluate multiple CNN architectures and training strategies with the aim of differentiating between DR-TB and DS-TB. We evaluate both pre-trained CNNs as simple N-layer custom CNNs. In terms of training strategies, we evaluate the use of different data augmentation approaches, augmenting the data statically beforehand or dynamically during training. Along with that, we generate synthesized images for DR-TB and DS-TB from the original images using Generative Adversarial Networks (GANs). We utilize a unique TB dataset provided by the US National Institute of Allergy and Infectious Diseases [4]. This patient based dataset includes clinical, genomic, and radiological data (CXRs and CT), but most importantly, it includes the results of drug susceptibility testing. Finally, we utilize several publicly available TB image datasets with unknown drug susceptibility to further enhance our classifier training.

## II. Previous Work

Computational identification and classification of lung diseases in medical images has been greatly facilitated by advancements in deep learning [5]. In the context of TB, usage of CXRs to classify an image as TB/not-TB has been described in multiple publications. Even simpler architectures such as AlexNet and GoogleNet, used with around 1000 training images, have shown good performance, exceeding 95% accuracy on some datasets [6]. The specific task of detecting TB in CXRs has seen great success, with multiple commercial products available, and a recent study reporting an area under the receiver operating characteristic curve of 0.92 or greater, when evaluated on unseen data [7].

Very few works have dealt with identifying the type of TB, DR-TB or DS-TB, from images. As part of the ImageCLEF 2017 and 2018 challenges, this question was posed using CT images. In 2017/2018 participants of the challenge were provided with a training set comprised of 230/259 training CTs and 223/236 testing CTs. After running the challenge for two years, the organizers said that “After two editions we concluded that the MDR (Multi-Drug Resistant) subtask was not possible based only on the image.” ^1^ It should be noted that the size of the training dataset was very small, and likely adversely affected deep learning based approaches.

In a different study [8], our group had moderate success in differentiating between DR-TB and DS-TB using CXRs, achieving an AUC of 0.66 utilizing hand-crafted shape and texture features. In clinical research, several publications describe using imaging (CXR or CT) to identify clinical findings that potentially differentiate between DR-TB and DS-TB. In [9], the authors found the DR-TB class to have more large lesions whereas the DS-TB class had more medium and small lesions. In [10], the authors found that the DR-TB class was characterized by having thick-walled cavities. Finally, in [11], the authors found that presence of multiple cavities was a predictor of DR-TB.

Based on our initial results, and the more recent clinical observations, we believe CXRs can potentially be used for differentiating DR-TB from DS-TB using a deep learning approach, which is described in the following sections.

## III. Methods

To discriminate between DR-TB and DS-TB, this work collects and processes CXR images from different sources, selects models trained with deep learning based approaches, and uses training strategies to improve classification performance. The CXRs used in this work are from the following sources: TB Portals [4], Montogomery County and Shenzhen chest X-ray sets [12], and the TBX11K large scale tuberculosis dataset [13]. Table I lists the number of samples for each set. The TB Portals dataset is the only one which contains results of drug susceptibility testing, indicating if the image is DR-TB or DS-TB. For all other datasets, we assume the images are DS-TB as that is significantly more common. To ensure that our evaluation is valid, we only use images from the TB Portals dataset in our testing.

### A. Data preprocessing

#### 1) Data Selection:

The TB portals dataset contains images from hospitals in 16 countries. Because of this, there are variations in the quality of images. We discarded images that are non-pulmonary, from lateral views, or non-grayscale.

Because an early-stage distinction of drug sensitivity or resistance is desirable, only images from a patient’s first visit were selected for this analysis. Further, to give equal weight to all patients, a single image was used per patient even when multiple images were acquired on that visit.

#### 2) Cropping of lung regions from CXRs:

**Fig. 1:**
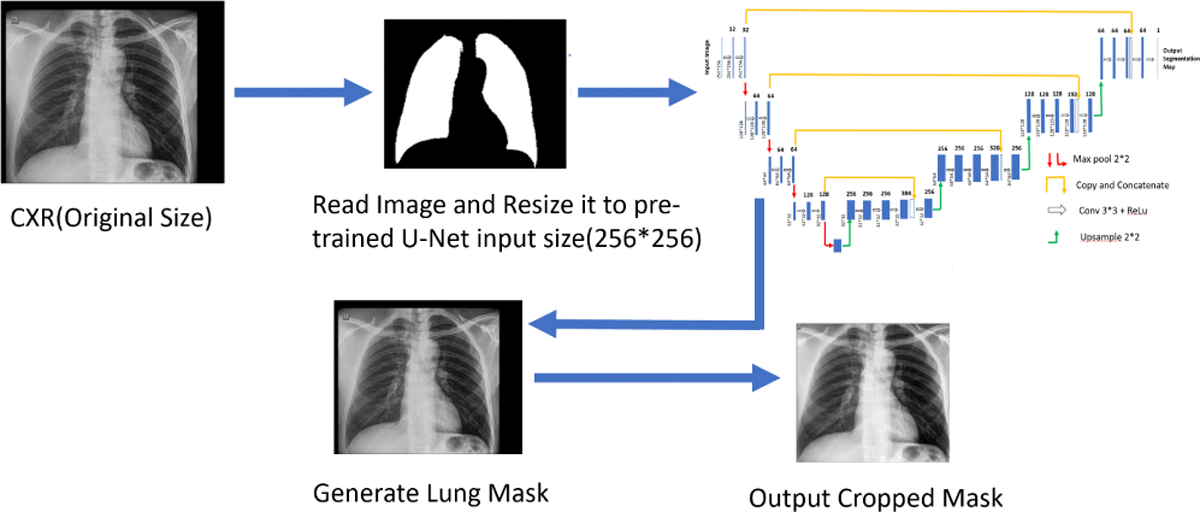
Preprocessing pipeline for CXRs

CXR images often include significantly sized regions that are outside the lungs, such as shoulders and neck. These regions are not relevant for the classification and in fact can be a hindrance in developing accurate models. Cropping a tight region around the lungs and removing unnecessary regions also allows for a more consistent size of the lungs across multiple images once they are rescaled. We therefore use a deep learning approach to crop the original CXRs to the lung region. During the cropping process, the CXRs are blurred by Gaussian smoothing with a standard deviation of 0.5 to reduce the high frequency signal components. Each smoothed image is resampled to a fixed dimension (256×256), before normalizing the intensities to zero mean and unit standard deviation. The CXRs are passed through a U-Net based segmentation model [14]. The resulting lung masks are used to compute a bounding box to crop the lung region from the original CXRs. The segmentation model was trained on two datasets [12], [15] yielding an IoU of 0.971 and 0.956, respectively. Subsequently, the original images are cropped using the bounding box coordinates, downsampled, and renormalized. Figure 1 illustrates this process.

### B. Network Architectures

For the classification task, we evaluate several standard CNN architectures as well as three custom CNNs. The standard networks include: AlexNet [16], DenseNet [17], InceptionV3 [18], ResNet [19], and Xception [20]. For each of the standard networks, we removed the dense layers after the final convolutional layer and added new dense layers. Table II shows the number of parameters for each of the networks.

### C. Data Augmentation

Most deep CNNs require a large amount of good quality data for the models to generalize well. As the number of available samples for each class is relatively small, we use two augmentation approaches:

#### 1) Image transformations:

The following transformations are applied to the original images: rotation (±10°), translation (±5 pixels), blurring (𝒩(0.0,1.0)), and histogram equalization. We intentionally apply small parameter values for these methods as they replicate the relatively small variations in X-ray images compared to images from other domains. We evaluate the usage of **static augmentation**, one time application of the transformations to the entire dataset before training starts, and **dynamic augmentation**, where original images are modified on the fly during batch training.

#### 2) Synthetic Image Generation:

Aside from image transformations, we synthesize images from both categories to increase the number of samples. We use the progressive growing of generative adversarial networks (PG-GANs) [21]. PG-GANs were chosen as they have been shown to generate relatively stable, quality, and variant images. For generating synthetic images for each category during each growth phase of [4×4, 8×8, . . . , 128×128], batch sizes and epochs of [128, 64, 64, 32, 32, 16] and [100, 250, 250, 250, 250] were used respectively. The final outputs are up-sampled to the input size of the classifying network.

## IV. Experiments

We evaluate model capabilities to distinguish between DR-TB and DS-TB on a patient-level basis. In all experiments, we use 10-fold cross validation.

We start by evaluating multiple models on the TB portals dataset using non-augmented training. We then evaluate the effects of various augmentation strategies on the best models. Finally, we add TB images from external sources, labeling all of them as DS-TB, to the best model from the last step.

### A. Model Selection

The pretrained network architectures were designed to address multi-class classification tasks. While we only deal with two classes (DR-TB and DS-TB), the size of the available dataset is much smaller in comparison. We therefore initially evaluate multiple standard architectures and several custom CNNs using the TB Portals dataset.

### B. Effects of Augmentation

Once we identify the more promising architectures, we explore the effects of dynamic and static augmentation strategies as well as utilizing synthetically generated images in the training stage. For this experiment, we only select the best performing pretrained-networks (InceptionV3 and Xception) and the best performing custom network. We also evaluate the effect of increasing the amount of statically augmented data on the balanced dataset created in the previous experiment.

### C. Including Additional Data

As shown in Table I, the number of DR-TB images in the TB portals dataset is significantly higher than the number of DS-TB images. In an effort to utilize images from all available patients in the imbalanced TB portals dataset, additional TB images from other sources were also included. We label these images as DS-TB, as this is the prevalent type of TB. The previous augmentation strategies were combined with the additional data to see if they influence the overall AUC performance. Note that these images are only used for training purposes as there is no drug susceptibility testing associated with them.

## V. RESULTS

In our network architecture comparison, without any augmentation, pretrained InceptionV3 and Xception networks had the best performance, as can be seen in Table II. These two networks, and several custom CNNs (6-layer, 10-layer, 12-layer), were also trained with random initialization. Among the custom networks, the 6-layer CNN had the best area under the ROC curve (AUC) with 0.74±.04 compared to the rest of the custom networks. When randomly initialized, the performance of InceptionV3 and Xception deteriorated.

Different augmentation methods and addition of synthetic images did not yield better performance for these networks, as shown in Table III. Performance did not scale with the increase in augmented data. When number of samples was increased to 3X and 4X original samples size by static augmentation, performance decreased. Interestingly, the performance of the custom 6-layer network improved with the same training strategy. We chose to continue our evaluation using the InceptionV3 network as its performance remained the most consistent with these augmentation strategies.

**Fig. 2:**
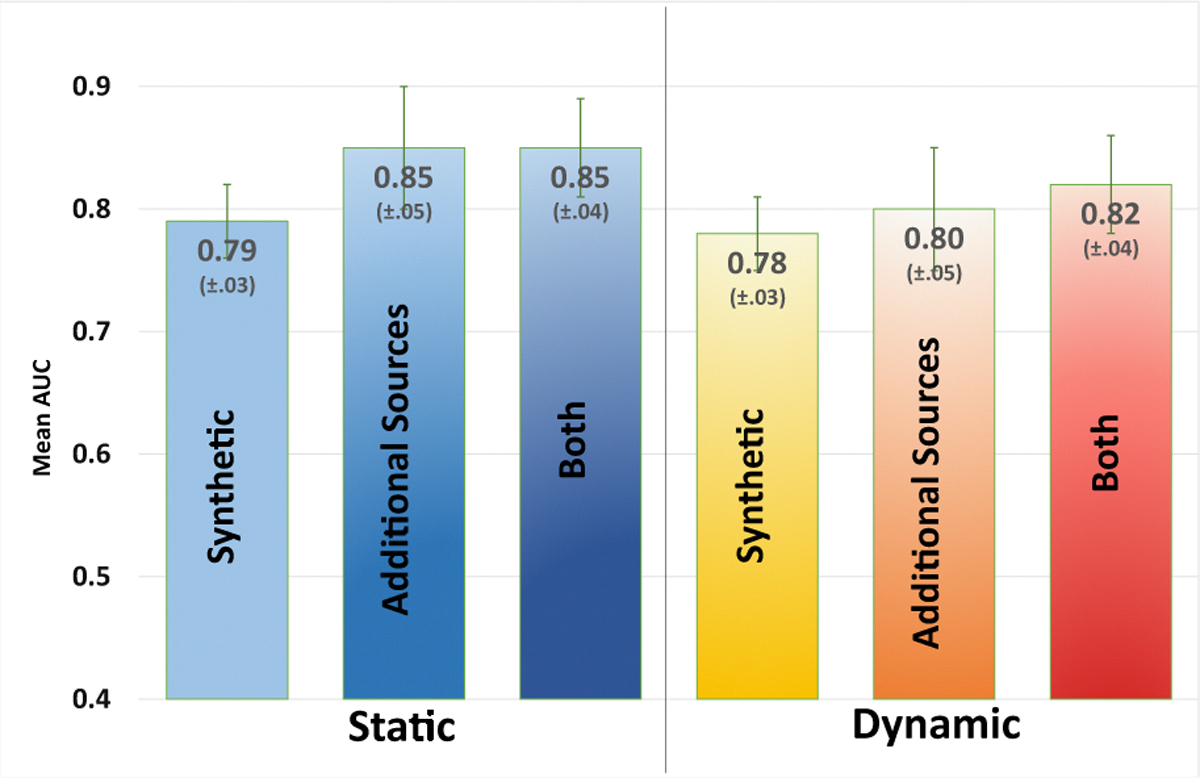
Mean AUC performances of the InceptionV3 network with *static* or *dynamic* augmentation and including a) *synthetic* images, b) images from [12] and [13] (referred in figure as *additional sources*) c) *both*. These additional images with static augmentation provided the best performance.

Figure 2 summarizes the performance evaluation of InceptionV3, using various datasets and augmentation strategies. We see that static augmentation has an overall positive effect on model performance compared to the dynamic augmentation strategy. We also see that the addition of images from other sources to the training set combined with static augmentation lead to the best AUC overall performance of 85%.

Although the inclusion of *both* the *synthetic* data and data from *additional sources* improves performance when using dynamic augmentation, it did not have an effect when using static augmentation. The variance in performance is slightly better when *both* synthetic and additional data are included.

**Fig. 3:**
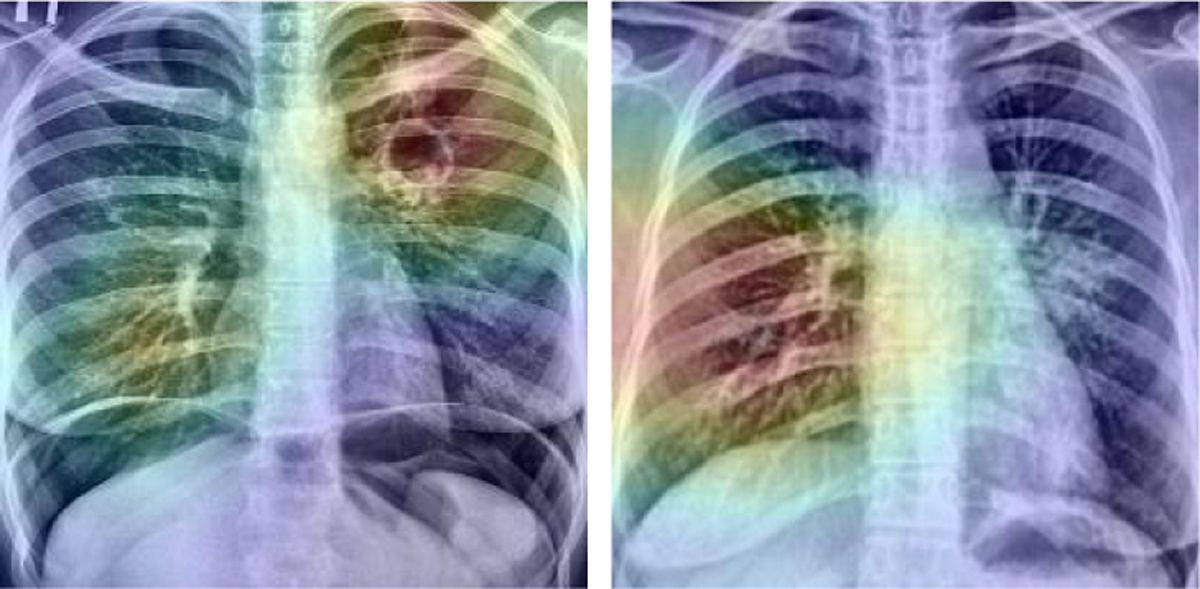
GradCAM heatmaps superimposed on the original images, Classified as DR-TB (left image) and DS-TB (right image) due to likelihood values of .99 and .05 respectively.

Finally, to inspire confidence in the predictions of our network, we utilize GradCAM heatmaps [22] to visualize its focus. Figure 3 shows two heatmaps for correctly predicted.

## VI. CONCLUSIONS

This paper presents an evaluation of models for discriminating between drug-resistant and drug-sensitive TB in the TB portals dataset, using augmentation strategies and other publicly available data. With a 10-fold cross validation, we achieve the best AUC performance of 85%. Even without augmentation and additional data, but with pretrained weights, we achieve a 81% AUC performance with InceptionV3 and Xception networks. GradCAM heatmaps affirm that the models learn from relevant areas from the CXRs during the training process. Despite discouraging earlier work in the literature, our work has shown that discriminating between DR-TB and DS-TB can be possible in CXRs for a sufficiently large training set.

## Figures and Tables

**TABLE I:** Number of images from each source.

Sources	DR-TB	DS-TB

TB Portals	1821	878
Montgomery County [12]*	0	58
Shenzhen [12]*	0	336
TBX11K [13]*	0	549
Synthetic (using GAN)	1000	1000

Total	2821	2821

* Patients from [12] and [13] are assumed to be drug sensitive.

**TABLE II:** Mean AUC (Area Under ROC Curve) of 10-fold cross validation results when various pretrained and custom networks are tested on TB Portals dataset

Architecture	Parameters (in millions)	AUC
Pretrained Networks		
AlexNet [16]	5.7	0.79 (± .02)
DenseNet121 [17]	7.2	0.79 (± .02)
DenseNet201 [17]	18.6	0.80 (± .02)
**InceptionV3** [18]	22.3	**0.81 (± .03)**
InceptionResNetV2 [18]	54.7	0.77 (± .05)
ResNet50 [19]	24.1	0.80 (± .03)
ResNet152 [19]	58.7	0.77 (± .03)
**Xception** [20]	21.3	**0.81 (± .02)**
Random initialization		
**6-layer CNN**	3.0	**0.74 (± .04**)
10-layer CNN	8.6	0.70 (± .03)
12-layer CNN	9.1	0.65 (± .04)
InceptionV3 [18]	22.3	0.76 (± .03)
Xception [20]	21.3	0.76 (± .03)

**TABLE III:** AUC with *dynamic* and *static* augmentation and with augmentation using GAN generated images.

Network Architecture	Dynamic	Static			Synthetic
		*2X*	*3X*	*4X*	

InceptionV3 (pretrained)	0.80 (±.03)	**0.81** (±.03)	0.80 (±.02)	0.79 (±.02)	**0.81** (±.02)
Xception (pretrained)	0.80 (±.03)	0.80 (±.03)	0.77 (±.04)	0.79 (±.03)	**0.81** (±.03)
6-layer CNN	0.76 (±.03)	0.76 (±.02)	0.74 (±.02)	0.76 (±.03)	0.75 (±.03)

## Footnotes

1
https://www.imageclef.org/2019/medical/tuberculosis/
